# Quality of life, social support and self-efficacy in women after a miscarriage

**DOI:** 10.1186/s12955-020-01662-z

**Published:** 2021-01-07

**Authors:** Grażyna Iwanowicz-Palus, Mariola Mróz, Agnieszka Bień

**Affiliations:** 1 Chair and Department of Development in Midwifery, Faculty of Health Sciences, Medical University of Lublin, Lublin, Poland; 2 Obstetrics and Gynecology Department and Clinic, Cardinal S. Wyszyński Regional Specialist Hospital, Lublin, Poland

**Keywords:** Miscarriage, Pregnancy loss, Quality of life, Social support, General self-efficacy

## Abstract

**Background:**

Pregnancy loss is typically experienced as a traumatic, critical event, which may lead to secondary psychological health disorders. Its burden involves both the experience of loss and related medical issues, which are associated with pain, hospitalization, limitation in one’s social roles, decreased sense of security, and changes in one’s perceived quality of life. The purpose of the present study was to evaluate levels of quality of life (QoL), social support and self-efficacy among women who had suffered a miscarriage.

**Methods:**

The study was performed using a diagnostic survey method with questionnaires administered to 610 patients hospitalized due to spontaneous pregnancy loss in hospitals in Lublin (Poland). The instruments used were: the Berlin Social Support Scales (BSSS), the Generalized Self-Efficacy Scale (GSES), the WHOQoL–BREF questionnaire, and a standardized interview questionnaire.

**Results:**

Respondents rated their overall quality of life (3.90 points) higher than their overall perceived health (3.66). In terms of social support, the highest scores were noted for perceived available instrumental support (M = 3.78), perceived available emotional support (M = 3.68) and actually received support (M = 3.60). The mean generalized self-efficacy score among the women after pregnancy loss was 30.29. Respondents’ QoL was significantly correlated with multiple social support subscales and self-efficacy (p < 0.05).

**Conclusions:**

Women after a miscarriage perceive their overall quality of life as better than their overall health, while reporting the poorest QoL in the psychological domain. They also have a high level of self-efficacy. Regarding the types of social support, perceived available support, both instrumental and emotional, and actually received support was rated highly. Social support and self-efficacy contributed to better perceived QoL among the respondents.

## Background

According to the World Health Organization (WHO), the term “fetal death” refers to the intrauterine death of a fetus at any time during pregnancy. The Royal College of Obstetricians and Gynecologists applies the term “miscarriage” to pregnancy losses occurring until 24 weeks of gestation [1]. In Poland, a miscarriage is defined as the loss of pregnancy before the 22nd week of gestation or when the weight of the dead fetus does not exceed 500 g. The incidence of miscarriage is correlated with gestational age, and is estimated at approx. 15%, out of which 80% occur within the first trimester. Recurrent miscarriages account for 1–2% of cases [1–3].

Pregnancy loss is typically experienced as a traumatic, critical event, which may lead to secondary psychological health disorders. This burden involves both the experience of loss and related medical issues, which are associated with pain, hospitalization, limitation in one’s social roles, decreased sense of security, and changes in one’s perceived quality of life (QoL) [4–10].

The process of coping with difficult situations is affected by a number of factors, including self-efficacy, defined as an individual’s belief about their ability to achieve their objectives in a particular life situation [11, 12]. Individuals with a low level of self-efficacy may focus on their deficiencies, and tend to experience strong emotions, anxiety or even depression. In turn, a high level of self-efficacy is positively correlated with optimism, satisfaction with life, and a healthy distance to life, as well as with increased capacity to overcome difficulties or stress, and lower stress levels [11–13]. Alongside one’s personal competences, sense of control and coherence, self-efficacy is another personal resource that influences the effectiveness of support [12–14].

Social support is defined as assistance available to an individual in difficult situations. Broadly defined, it refers to issues of social integration and interpersonal relationships that have an impact on the individual, allowing them to feel surrounded by people upon whom they can rely. Being with another in a problematic situation reduces stress and makes one feel more secure and in control. Social support is a significant contributor to the maintenance of health, prevention of disease, and success of treatment. It is also a factor recognized as positively affecting an individual’s QoL [15].

The communication between medical personnel and a patient who has lost a pregnancy is a special kind of social interaction, involving an exchange of emotions and instruments for action. Recognizing a hospitalized patient’s individuality and complexity has a positive impact on their relationship with medical personnel and contributes to greater care effectiveness and a lower incidence of psycho-emotional disorders associated with one’s health situation [16].

In modern medicine, the objective is not just to treat patients but also to improve their well-being and QoL. Hence the focus on achieving extramedical objectives within the treatment process, to help patients function normally not only in the physical domain but also in the psychological and social ones.

In medical science, research on QoL and social support provides knowledge on institutional efforts that need to be undertaken for the sake of patients, and on ways of supporting and empowering patients in their difficult circumstances [16]. This reflects a holistic approach to the subject of care and helps identify factors with the most significant impact. Such research contributes to the continued improvement of standards of care in specific conditions, and should therefore be integrated with treatment [17].

Considering the impact and complexity of pregnancy loss, with its multiple health-related, psychosocial and economic consequences, the present study aimed to evaluate the levels of QoL, social support and self-efficacy in women who had experienced a miscarriage.

## Methods

### Study design and population

The study was performed between August 2016 and February 2019, and included 610 patients hospitalized due to spontaneous pregnancy loss (until 22 weeks of gestation) in the following hospitals in Lublin, Poland: the Independent Public Teaching Hospital no. 1, the Independent Public Teaching Hospital no. 4, the Cardinal Stanisław Wyszyński Regional Specialist Hospital, and the John of God Independent Public Regional Hospital. The inclusion criteria were as follows: loss of a singleton pregnancy up to 22 weeks, consent to participate in the study, age above 18 years, suitable timing of the survey (3–6 days after the miscarriage, on the last day of hospitalization, once treatment completion has been ascertained), the patient being in good physical condition, and lack of psycho-physical disorders. Patients undergoing psychotherapy or psychiatric treatment, and patients in a poor psychological condition were excluded.

### Data collection

The survey questionnaire was administered to each patient on the last day of her hospitalization. Before communicating with each patient, information was obtained from the medical personnel regarding the stage of treatment, duration of hospitalization, and the psychological and physical condition of the patient. The study was approved by the Bioethics Committee of Lublin Medical University (KE-0254/221/2016) and by the managers and department heads in each hospital where the study was performed. Respondents were informed that their participation in the survey was anonymous and strictly voluntary, and that the results would only be used for research purposes. To ensure the highest possible quality and reliability of our study, information on the stage of treatment, duration of hospitalization, and the clinical condition of the patient was obtained from medical personnel before contact with each patient. The study instrument was given to each patient on the last day of her hospitalization (3–6 days of hospitalization), having ascertained that her treatment had been completed and her psycho-physical condition had been stabilized. Out of the 645 patients recruited for the study, 610 returned fully and correctly completed questionnaires, and were included in subsequent statistical analyses. The response rate was 94.57%.

### Assessments

The study used a diagnostic survey with questionnaires. The instruments used were: the WHOQoL–BREF questionnaire, the Berlin Social Support Scales (BSSS), the Generalized Self-Efficacy Scale (GSES), and a standardized interview questionnaire.

### Instruments

The World Health Organization Quality of Life Test-Bref (WHOQoL-BREF) is used to determine a patient’s QoL profile in the physical, psychological, social and environmental domains, as well as overall QoL and perceived health. Respondents rate 26 items on a scale from altogether negative to altogether positive. The scoring is positive, i.e. higher scores indicate a better QoL. The questionnaire’s internal consistency coefficient (Cronbach’s α) is 0.54–0.91 for individual domains; for the whole scale, it is 0.92 in healthy individuals and 0.95 in ill individuals [18, 19].

The Berlin Social Support Scales (BSSS) is a set of independent instruments measuring the behavioral and cognitive dimensions of social support: available and received support, protective buffering, need for support, and support seeking. Respondents rate each item on a scale of 1–4, where 1 indicates complete disagreement and 4 complete agreement with the statement. Higher scores indicate more social support. In the present study, results are presented as means for each scale. The Cronbach’s α for the questionnaire is 0.80 [20].

The Generalized Self-Efficacy Scale (GSES) is an instrument measuring the strength of an individual’s belief in their capacity to overcome difficulties and obstacles. Respondents rate 10 statements on a scale of 1 to 4 (*1–disagree, 2–somewhat disagree, 3–somewhat agree, 4–agree*). The total score, converted into standardized sten units, reflects the overall level of self-efficacy. Low self-efficacy is indicated by scores of up to 24 points (sten 1–4), moderate—by scores between 25 and 29 points (sten 5–6), and high—by scores of 30 or more points (sten 7–10). Cronbach’s α for the instrument is 0.85 [11, 21].

Standardized interview questionnaire used to collect respondents’ characteristics—having children, history of pregnancy loss, age, education, residence, relationship status, self-reported financial standing and professional activity (respondents in our study entered their profession in the survey questionnaire. Based on their responses, we have classified their professional activity as blue-collar work or white-collar work—a significant distinction in terms of physiology and ergonomics).

### Statistical analysis

Statistical analysis of the material was performed using IBM SPSS Statistics (v. 21) software. Quantitative variables were described using mean (M), median (Me), standard deviation (SD), and minimum (Min) and maximum (Max) values. For qualitative variables, numbers and percentages in each category were reported. A series of regression analyses were also performed using the enter method, with the physical, psychological, social and environmental domains of the WHOQOL-BREF as the dependent variables. The independent variables included: GSES score, perceived emotional support, perceived instrumental support, need for support, support seeking, and actually received support. The study used a significance threshold of *p* < 0.05.

## Results

Table 1 shows respondents’ characteristics. Most were women aged between 26 and 30 (32.6%), having completed college/university education (61.1%), living in province capitals (47.7%), married (80.5%), performing white-collar work (48.7%), and reporting a good socio-economic standing (60.8%). Moreover, most respondents had had children before (59.0%), and had miscarried for the first time (59.2%).

**Table 1:** Participants’ characteristics

Participants’ characteristics	*n*	%
Age		
< 25 y/o	77	12.6
26–30 y/o	199	32.6
31–35 y/o	195	32.0
> 35 y/o	139	22.8
Education		
Other than college/university	237	38.9
College/university	373	61.1
Residence		
Urban—province capital	291	47.7
Urban—other	116	19.0
Rural	203	33.3
Relationship status		
Married	491	80.5
Single	119	19.5
Professional activity		
Professionally inactive	135	22.1
White-collar work	297	48.7
Blue-collar work	178	29.2
Self-reported financial standing		
Very good	93	15.2
Good	371	60.8
Moderate	146	24.0
Having children		
No	250	41.0
Yes	360	59.0
History of pregnancy loss		
First pregnancy loss	361	59.2
1 previous pregnancy loss	175	28.7
≥ 2 previous pregnancy losses	74	12.1

The mean overall QoL score was 3.90 ± 0.77, and the mean overall perceived health score was 3.66 ± 0.76. In terms of specific domains, QoL was highest in the social domain (17.04 ± 2.54), and lowest in the psychological domain (14.91 ± 2.45). The highest scores on the social support scales were obtained for perceived available instrumental support (3.78 ± 0.43), and the lowest for protective buffering (1.89 ± 0.68). The mean generalized self-efficacy (GSES) score among the women who had experienced a miscarriage was 30.29 ± 4.01 (Table 2).

**Table 2:** Quality of life, social support and self-efficacy scores among women who had experienced a miscarriage

	M	Me	SD	Min	Max
QoL					
Overall quality of life	3.90	4.00	0.77	1.00	5.00
Perceived general health	3.66	4.00	0.76	1.00	5.00
Physical domain	15.96	16.00	2.14	8.57	20.00
Psychological domain	14.91	15.33	2.45	6.67	20.00
Social relationships domain	17.04	17.33	2.54	4.00	20.00
Environment domain	15.90	16.00	2.04	8.50	20.00
BSSS subscales					
Perceived available emotional support	3.68	3.75	0.44	1.00	4.00
Perceived available instrumental support	3.78	4.00	0.43	1.00	4.00
Need for support	3.16	3.25	0.57	1.00	4.00
Support seeking	3.09	3.20	0.66	1.00	4.00
Actually received support	3.60	3.80	0.40	1.00	4.00
Protective buffering	1.89	1.83	0.68	1.00	4.00
GSES	30.29	30.00	4.01	17.00	40.00

*M* mean, *SD *standard deviation, *Me* median, *QoL* Quality of Life, *BSSS* Berlin Social Support Scales, *GSES* Generalized Self-Efficacy Scale

Table 3 shows the results of regression analysis for the specific QoL (WHOQOL-BREF) domains, social support (BSSS), and generalized self- efficacy (GSES). QoL in the physical domain is positively correlated with perceived available instrumental support (*ß* = 0.152, *p* < 0.001) and self-efficacy (*ß* = 0.280, *p* < 0.001). The model that was tested accounted for 12% of variance in the physical QoL score.

**Table 3:** Regression analysis for WHOQOL-BREF, BSSS, and GSES scores in women after a miscarriage

WHOQOL-BREF domains																
Predictor	Physical health *F* = 39.027;* df* = 2;* p* < 0.001				Psychological *F* = 42.469*; df* = 5;* p* < 0.001				Social relationships *F* = 79.518;* df* = 4;* p* < 0.001				Environment *F* = 36.715;* df* = 5;* p* < 0.001			
	*B*	*ß*	*t*	*p*	*B*	*ß*	*t*	*p*	*B*	*ß*	*t*	*p*	*B*	*ß*	*t*	*p*
Constant	8.599		9.712	< 0.001	2.971		2.488	0.013	1.438		1.316	< 0.001	5.143		5.400	< 0.001
*BSSS Subscales*																
Perceived available emotional support	− 0.033	0.568	− 0.023	0.428	1.299	0.235	5.918	< 0.001	1.199	0.209	5.727	< 0.001	0.705	0.153	2.784	0.006
Perceived available instrumental support	0.753	0.152	3.947	< 0.001	0.027	0.632	0.020	0.385	0.100	0.058	0.077	0.386	0.604	0.128	2.230	0.026
Need for support	− 0.050	0.221	− 0.050	0.879	− 0.362	− 0.084	− 2.221	0.027	0.015	0.674	0.017	0.859	− 0.290	− 0.081	− 2.097	0.036
Support seeking	− 0.068	0.095	− 0.068	0.879	0.066	0.184	0.054	0.498	0.023	0.521	0.026	0.862	0.023	0.647	0.019	0.496
Actually received support	0.018	0.683	0.017	0.758	0.672	0.110	2.791	0.005	2.655	0.419	11.367	< 0.001	0.688	0.135	3.285	0.001
Protective buffering	− 0.071	0.070	− 0.074	0.955	− 0.288	− 0.079	− 2.156	0.031	− 0.285	− 0.076	− 2.185	0.029	− 0.054	0.152	− 0.058	0.903
GSES	0.149	0.280	7.239	< 0.001	0.212	0.348	9.334	< 0.001	0.072	0.113	3.355	0.001	0.142	0.281	7.454	< 0.001
Regression equation	Y = 8.599 + 0.149 × GSES + 0.753 × BSSSI Instr				Y = 2.971 + 0.212 × GSES + 1.299 × BSSSI Emo − 0.362 × BSSSII + 0.672 × BSSSIV − 0.288 × BSSSV				Y = 1.438 + 0.072 × GSES + 1.199 × BSSS Emo + 2.655 × BSSSIV − 0.285 × BSSSV				Y = 5.143 + 0.142 × GSES + 0.705 × BSSS Emo + 0.604 × BSSS Instr – 0.290 × BSSSII + 0.688 × BSSSIV			

*WHOQOL-BREF* The World Health Organization Quality of Life-Test Breif, *BSSS* Berlin Social Support Scales, *GSES* Generalized Self-Efficacy Scale, *BSSS I Emo* Perceived Available Emotional Support, *BSSS I Instr* Perceived Available Instrumental Support, *BSSS II* Need for Support, *BSSS III* Support Seeking; *BSSS IV* Actually Received Support, *BSSS V* Protective Buffering Support

The psychological QoL domain was analyzed next. This factor was positively correlated with perceived available emotional support (*ß* = 0.235, *p* < 0.001), actually received support (*ß* = 0.110, *p* = 0.005), and self-efficacy (*ß* = 0.348, *p* < 0.001). Negative correlations were found with need for support (*ß* = − 0.084, *p* < 0.027) and protective buffering (*ß* = − 0.079, *p* = 0.031). This model accounted for 26% of variance in the psychological QoL score.

Analyses demonstrated that: perceived available emotional support (*ß* = 0.209, *p* < 0.001), actually received support (*ß* = 0.419, *p* < 0.001), protective buffering (*ß* = − 0.076, *p* = 0.029), and self-efficacy (*ß* = 0.113, *p* < 0.001) were significantly correlated with the social QoL domain. The model accounted for 35% of variance in the social QoL score.

QoL in the environmental domain was positively correlated with perceived available emotional (*ß* = 0.153, *p* = 0.006) and instrumental support (*ß* = 0.128, *p* = 0.026), actually received support (*ß* = 0.135, *p* = 0.001), and self-efficacy (*ß* = 0.281, *p* < 0.001). A negative correlation was found with need for support (*ß* = − 0.081, *p* = 0.036). The model accounted for 23% of variance in the environmental QoL score (Table 3).

## Discussion

In modern medicine, the objective is not just to treat patients, but also to improve their well-being and QoL, hence the increasing interest in research on social support and QoL experienced by patients with a variety of conditions. Such a comprehensive assessment is especially important in patients who have lost a pregnancy, as this experience entails a number of consequences, not only physical, but also psychological and social, and at times, even financial. Each patient has her own needs and beliefs that affect her individual perception of her health and living situation. Therefore, we undertook to evaluate the levels of QoL, social support, and self-efficacy in this patient group [16, 17].

Our findings are to some extent consistent with those reported by other researchers studying the subject. Like patients with hyperglycemia in pregnancy and those treated for polycystic ovary syndrome, the women who have experienced a miscarriage who were studied rated their overall QoL higher than their overall perceived health [22, 23].

The social relationships domain was scored highest, as was the case in studies by Couto et al. and Tavoli et al. regarding women after pregnancy loss [24, 25]. The high scores in the physical domain were also consistent with the report by Tavoli et al. [25]. Interestingly, in the QoL self-assessment by physically active women during uncomplicated pregnancy, the scores in the physical domain were next to lowest, while the highest scores were reported in the psychological domain [26]. In the present study, women who had lost a pregnancy reported lower QoL in the psychological domain. Respondents’ psychological well-being was also rated lower than other QoL aspects in the studies by Couto et al. and Tivoli et al., although these authors placed more emphasis on the limitations in the performance of social roles, associated with emotional difficulties [24, 25]. In turn, Song et al., analyzing the long-term effects of a child’s death on the parents, reported much poorer QoL than in the case of non-bereaved individuals [27]. Other authors also highlighted considerable differences in QoL between pregnant subjects with no history of obstetric problems and ones after a pregnancy loss, with the latter group having lower QoL [24, 25, 28, 29].

QoL is also significantly associated with social support, which is a major contributor to a patient’s health and treatment success [15]. In the group of women who have miscarried, perceived available support, both instrumental and emotional, and actually received support was rated highly. The present statistical analysis findings are comparable to those reported by Iwanowicz-Palus G. et al. in a group of patients with hyperglycemia in pregnancy [22].

The association between social support and satisfaction with life was previously demonstrated e.g. by Strine et al. in their US study. They found that overall dissatisfaction with life increased as the level of social support decreased [30]. Gul et al. [31], Emmanuel et al. (2012) and Shishehgar et al. [33] also reported a positive correlation between social support and QoL in pregnant women [31–33].

A literature review demonstrates that social bonds have a beneficial impact on various aspects of an individual’s psycho-physical condition, including reduced health-related stress, anxiety, and depressive symptoms [30, 33–37]. The results of the present study indicate an association between perceived available emotional support and actually received social support, on the one hand, and better QoL in the psychological domain on the other. The quality of social support received from various sources and the professional demeanor of medical personnel also affect the patient’s psychological well-being and satisfaction with life.

Pregnancy loss is a stressful event that may prompt a psychological crisis [7, 9]. The process of coping with a difficult health-related situation is shaped by a number of factors, one of which is self-efficacy [11]. A literature review does not reveal many reports on the subject in the field of gynecology. It is, however, a highly consequential topic, as an individual’s expectations and beliefs have a significant impact on their actions, as well as on their physical and psychological health [11, 13, 38].

The patients who had experienced a miscarriage and who participated in our survey had a higher level of generalized self-efficacy than that found in a standardization group for the Polish population, which was moderate (this included women with pregnancy complications, post-menopausal women, diabetic patients, patients on dialysis, and patients with a history of myocardial infarction) [11]. This suggests that the patients studied here were involved in their treatment process and actively coping with their difficult situation. Similar results were found in groups of patients who had had a mastectomy and pregnant women with hyperglycemia [11, 22].

Self-efficacy may affect QoL or satisfaction with life. It is also one of the cognitive factors that affect the way individuals manage stress [11–13, 19].

The available publications suggest that building self-efficacy by strengthening a patient’s sense of control and ability to manage a given life situation may counteract negative emotions or exacerbations of depressive symptoms [39–42]. An analysis by Nikcevic et al. demonstrated a significant association between more self-efficacy and less anxiety and depression in women who had lost a pregnancy [38]. The present analysis confirms the impact of self-efficacy on all QoL domains in the women studied, and the strongest positive correlation was found for the psychological QoL domain.

As pregnancy loss may have negative psychological health consequences, it is worth considering strategies to enhance self-efficacy, especially among women found to have lower levels of this resource [5, 8–10]. The present findings may contribute to a better understanding of care involving not just professional medical interventions, but also the provision of adequate social support. Providing support and improving the self-efficacy of women after pregnancy loss seem extremely important for patients’ psycho-social well-being and QoL, on a level with effective medical management. In practice, these aspects should be included among the priorities of medical personnel’s daily work.

### Strengths and limitations of the study

The strengths of our study include the sample size (610 patients) and personal communication with each respondent. As we used standardized instruments, other researchers interested in issues related to pregnancy loss will be able to compare results, continue in-depth research and monitor changes. The available studies on women who have had a miscarriage were typically performed weeks or months after the event. We investigated patients during hospitalization, as this is when the coping process typically begins.

One difficulty was associated with the personal administration of surveys to the respondents, as during their 3–6 days of hospitalization, they also underwent multiple intensive diagnostic and treatment procedures. Therefore, to ensure the highest possible quality and reliability of our study, information on the stage of treatment, duration of hospitalization, and the clinical condition of the patient was obtained from medical personnel before contact with each patient. The study instrument was given to each patient on the last day of her hospitalization, having ascertained that her treatment had been completed and her psycho-physical condition had been stabilized.

One limitation of the present study is the lack of data on the QoL of women with a normal pregnancy course until week 22, against which we could compare the scores found in women after pregnancy loss.

Our study is limited by its cross-sectional design, as it does not allow for identifying any causal relationships between quality of life, social support and self-efficacy in women who have had a miscarriage.

## Conclusions

1. Women who have miscarried rate their overall quality of life higher than their overall perceived health. In terms of specific domains, their QoL is highest in the social domain, and lowest in the psychological domain.
2. Among the various types of social support, perceived available support, both instrumental and emotional, and actually received support was rated the highest.
3. Women after a miscarriage have a high level of generalized self-efficacy.
4. Social support and self-efficacy affects the perceived quality of life of women who have had a miscarriage.

## Data Availability

Data from this study will be shared with qualified investigators upon reasonable request for scientific purposes.

## Abbreviations

BSSS: Berlin Social Support Scales
BSSS I Emo: Perceived available Emotional Support
BSSS I Instr: Perceived available Instrumental Support
BSSS II: Need for Support
BSSS III: Support Seeking
BSSS IV: Actually Received Support
BSSS V: Protective Buffering Support
GSES: Generalized Self-Efficacy Scale
M: Mean
Me: Median
QoL: Quality of Life
SD: Standard deviation
WHOQOL-BREF: The World Health Organization Quality of Life-Test Bref
